# Electromembrane Processes: Experiments and Modelling

**DOI:** 10.3390/membranes11020149

**Published:** 2021-02-20

**Authors:** Luigi Gurreri, Alessandro Tamburini, Giorgio Micale

**Affiliations:** Dipartimento di Ingegneria, Università degli Studi di Palermo, viale delle Scienze Ed. 6, 90128 Palermo, Italy; luigi.gurreri@unipa.it (L.G.); giorgiod.maria.micale@unipa.it (G.M.)

This Special Issue of *Membranes* journal focuses on electromembrane processes and is motivated by the increasing interest of the scientific community towards their characterization by experiments and modelling for several applications.

The increasing demand of water and energy poses technological challenges for the implementation of efficient and sustainable concepts. In this perspective, electromembrane processes can play a crucial role in green chemistry schemes oriented towards circular economy approaches and renewable energy systems. Electromembrane processes are based on the use of ion-exchange membranes under the action of an electric field. Versatility, selectivity, high recovery, and chemical-free operations are their main advantages. Experimental campaigns and modelling tools are prompting the improvement of consolidated processes and the development of novel concepts. Moreover, hybrid and integrated systems offer synergistic benefits for efficiency enhancement. Several application fields have been proposed (in chemical, food, pharmaceutical industries and others) including desalination, water and wastewater treatment, recovery of valuable products, concentration and purification operations, chemical production, energy conversion and storage. Significant advancements have been achieved in recent years, and, currently, research is very active in this field.

In this Special Issue, we have collected contributions on recent advancements of electromembrane processes and their applications, with a focus on the development of cutting-edge engineered systems by experiments and/or models. The Special Issue contains eight articles. One review and three research articles regard electrodialysis, two research articles pertain to reverse electrodialysis, one research article focuses on an integrated membrane process including electrochemical intercalation–deintercalation, and a perspective article regards acid-base flow battery.

Electrodialysis (ED) is a mature technology that has been studied for a variety of applications. Among them, the treatment of wastewater has, in the last few years, attracted very broad attention.

In this Special Issue, Gurreri et al. [1] present the first comprehensive review of studies on ED applications in wastewater treatment for environmental protection and resources recovery, outlining the current status and the future prospect. About 400 relevant higher-quality scientific papers are reviewed and discussed. ED treatments of effluents from various industrial processes, municipal wastewater or saltwater treatment plants, and animal farms, are considered. The review shows that ED and unconventional configurations of ED, i.e., bipolar membrane electrodialysis (BMED), selectrodialysis (SED), electrodialysis metathesis (EDM) and electrodeionization (EDI), have great potential in desalination and valorization strategies of wastewater for a broad range of applications to recover water and/or other valuable components. The main ones are metals, salts, acids and bases, nutrients, and organics. Energy recovery via reverse electrodialysis (RED) is another possibility. The large variety of uses of conventional ED and similar technologies is discussed by analyzing experimental results, process performance, strengths and drawbacks, and techno-economic competitiveness. Therefore, conclusions and outlook are provided, highlighting the main technical challenges, the current status of the process scale in the various applications, and the key points for future R&D. Recent advances and emerging applications are reviewed, along with examples among the few well-established implementations in real environments. The review shows that recent research efforts have led to the development of several enhanced or novel systems, with the possible implementation of techno-economically affordable and competitive (near) zero liquid discharge approaches. Few real plants have been installed due to techno-economic challenges that are still present. Overall, research is opening new routes for the large-scale use of ED techniques in a plethora of treatment processes.

Sheng et al. [2] prepare ZSM-5 zeolite/PVA (polyvinyl alcohol) mixed matrix cation-exchange membranes with high monovalent permselectivity and test them for recovery of either acid or Li^+^. Zeolites are crystalline aluminosilicates with pores and cavities of molecular dimensions and have excellent ion-exchange capacity. Commercial low-price ZSM-5 zeolite with 0.5–0.6 nm pore size is mixed at various fractions with PVA for the casting procedure. The prepared membranes are tested for H^+^/Zn^2+^ and Li^+^/Mg^2+^ selective separation for possible application of ED for acid recovery and saltwater desalination with scaling prevention, respectively. The optimal content of ZSM-5 zeolite of 50 wt% leads to higher permselectivity values compared to other commercial and non-commercial membranes, i.e., $P_{Zn^{2+}}^{H^{+}}$ = 34.4 and $P_{Mg^{2+}}^{Li^{+}}$ = 3.7. This result is achieved thanks to the transport facilitated for protons and small ions by the ZSM-5 sites and to the transport hindered for larger hydrated cations by the microporous crystalline morphology. The SEM analysis shows a uniform dispersion and the XRD patterns exhibits identical characteristic peaks as the pristine ZSM-5 powder. As the amount of ZSM-5 is increased, water uptake and area swelling decrease. Finally, the prepared membranes exhibit high limiting current density and low electrical resistance, thus being very attractive for practical applications.

Uzdenova and Urtenov [3] perform 2-D Direct Numerical Simulations to compare current–voltage curves and parameters of the electroconvective vortex layer in the potentiodynamic and galvanodynamic regimes for an ED dilute channel with forced flow in contact with homogeneous membranes [3]. In this case, electroconvection arises due to uneven distribution of concentration along the channel, which generates a tangential component of electric field. The Nernst–Planck–Poisson and Navier–Stokes equations are solved. The computational domain consists of half channel in contact with the CEM surface. The channel thickness, the channel length, the inlet concentration, and the mean velocity are of 500 µm, 1 mm, 0.1 mol m^−3^ and 3.8 mm s^−1^, respectively. The model is implemented by the finite element COMSOL Multiphysics^®^ commercial platform. Realistic *i*–*V* curves are predicted, exhibiting four current regimes, i.e., underlimiting, limiting, overlimiting, and chaotic overlimiting. The potentiodynamic and galvanodynamic modes have similar behavior, apart from the oscillations of current and voltage, respectively, in the chaotic overlimiting region. The limiting–overlimiting and overlimiting–chaotic overlimiting transitions are characterized by hysteresis. Vortices rotating in the same direction are developed in the overlimiting region. Instead, large complexes of several counter-rotating vortices are formed in the chaotic overlimiting regime. The vortex layer has length and thickness increasing as the applied current or voltage increase, and the expansion rate is greater in the range close to the limiting current. This is the range in which the vortex density per unit length decreases up to a minimum, but it then increases with the development of large vortex complexes.

The ion transport at overlimiting currents is also studied via mathematical modelling by Urtenov et al. [4] in order to investigate the formation and properties of the local maximum (or minimum) space charge. Two systems are considered: the depleted solution at a CEM, and a desalination channel between a CEM and an AEM. A 1-D dynamic modelling based on the Nernst–Planck–Poisson equations is performed. At overlimiting currents, the counter-ion concentration decreases in the boundary layer and in the extended space charge region up to a minimum value before the electric double layer at the membrane interface. At this point, the space charge has a non-zero local minimum, whose value depends on the applied voltage. Moreover, the space charge has a local maximum at a certain point between the electroneutral solution and the point of the local minimum. In the simulations, as the imposed cation concentration at the CEM increases, the local maximum of space charge moves towards the membrane until it disappears together with the local minimum. This result shows that the local values of charge density exist due to the limited ion-exchange capacity of the membranes. In a transient system with increasing applied voltage, the local maximum moves similarly to a single soliton-like wave into the solution but changes slowly its size and shape. In the desalination channel, a different behavior is observed. In the case of a KCl solution, two waves of opposite charge move towards each other and interact up to breakdown. As the voltage increases, new waves are not formed, since the solution concentration is practically zero. With an NaCl solution, the difference was that the negative peak is generated later or even does not arise, depending on the applied linear growth rate of voltage.

Reverse electrodialysis (RED), which is the opposite process with respect to ED, converts the mixing free energy of two streams with different salt concentration into electrical energy. RED systems have been widely studied in the last decade. However, its development is still limited to prototypal installations, due to low power density and low membrane performance/durability with natural solutions, as well as high membrane cost. Therefore, several studies are currently performed to improve the process performance regarding different aspects.

Merino-Garcia et al. [5] perform a monolayer surface modification to functionalize heterogeneous Ralex Anion-Exchange Membranes with poly(acrylic) acid (PAA) yielding an increased monovalent permselectivity. After a contact of 24 h with the PAA aqueous solution, the modified AEMs are characterized by measuring contact angle, water uptake, ion exchange capacity, fixed charge density, and swelling degree. The electrochemical characterization is performed by cyclic voltammetry and electrochemical impedance spectroscopy. Permselectivity and fouling behavior are evaluated via mass transport experiments. The modified membranes show a significantly enhanced monovalent permselectivity and hydrophilicity, by increasing sulfate rejection by 36–54% and decreasing the contact angle by 15–31%. Higher current responses are also observed, while the electrical resistance exhibits only a small increase. However, in the presence of humic acid, used as model organic foulant, the overall ion transport and the monovalent selectivity of the modified membranes are reduced. Therefore, the technical feasibility of the proposed modification, which may improve the RED process efficiency, is demonstrated, but fouling mechanisms and appropriate pre-treatments and cleaning strategies should be studied.

Jalili et al. [6] present a Computational Fluid Dynamics (CFD) study on reverse electrodialysis. Single channels, 12.6 mm long with staggered hemi-circular or triangular non-conductive spacers, are simulated in two dimensions. Numerical simulations are based on the Navier–Stokes and Nernst–Planck governing equations under electroneutrality assumption, computing the pressure, velocity, concentrations, and electric potential fields. The open circuit voltage, the cell pair resistance and the peak power density are calculated. By including the pumping power losses, the net power density is computed. The freeware open source OpenFOAM platform (finite-volume method) is used. A factorial design parametrical study is performed with a total of 2 × 2^4^ test cases, given by two values for each of the four quantities, i.e., velocity, temperature, corrugation density and height for each of the two corrugation shapes. Results show that the temperature is the most influential parameter, leading to an increase of 43% in the net peak power density when passing from 25 to 55 °C. In descending order, lower effects are provided by inlet velocity, corrugation density, and corrugation height. The corrugation shape does not affect significantly the producible maximum peak power density. The efficiency of using low-grade waste heat to increase the feed temperature should be evaluated in future works.

The integration of electromembrane processes with pressure driven membrane processes can offer several solutions in water treatment for different aims.

Xu et al. [7] propose an integrated treatment process of brine with a high Mg^2+^/Li^+^ mass ratio for preparing Li_2_CO_3_, which is an important raw material. The process included electrochemical intercalation–deintercalation, nanofiltration, reverse osmosis, evaporation, and precipitation. The electrochemical intercalation–deintercalation (EID) method is used to maximize the separation between magnesium and lithium. EID is performed by a two-chamber electrolytic cell separated by an AEM, with LiFePO_4_ anode and FePO_4_ cathode (17 × 20 cm^2^). The anode chamber is filled with a 5 g L^−1^ NaCl solution as supporting electrolyte, the cathode chamber with brine (2.05 g L^−1^ Li^+^, 120.56 g L^−1^ Mg^2+^, 360.9 g L^−1^ Cl^−^ etc.). Lithium of the LiFePO_4_ anode intercalates to the supporting electrolyte, and lithium of the brine intercalates into the FePO_4_ cathode, while chloride migrates through the membrane from the catholyte to the anolyte. The EID method can thus extract lithium by subsequent cycles. The EID experiments show excellent separation performance, by reducing the Mg^2+^/Li^+^ mass ratio from 58.5 (brine) to 0.93 in the anolyte with 90.6% recovery of lithium after the second cycle, and low concentration of impurities. Nanofiltration and reverse osmosis are used for purification (removal of residual magnesium and other multivalent ions) and concentration (lithium enrichment) of the EID anolyte. After evaporation (electric furnace) and further removal of magnesium (precipitation with NaOH), industrial-grade Li_2_CO_3_ is prepared via chemical precipitation. The direct recovery of lithium from the brine to produce Li_2_CO_3_ is ~70%, but a higher recovery would be feasible in a system with recirculation of the solutions.

Systems based on reversible electromembrane processes may be used for energy storage, which is crucial for a greater penetration of renewable energies. In principle, a single unit may be employed and operated as ED process (charging mode) to separate a saline solution into a concentrate solution (when an energy surplus is available) and operated as RED (discharge mode) to recover energy from the controlled mixing of the two solutions (when energy demand is high). Unfortunately, although the significantly low environmental impact, the resulting battery named concentration gradient flow battery has a low energy density. In this respect, the coupling of bipolar membrane ED and RED may offer a sustainable solution due to acceptable values of energy/power density (about 10 kWh m^−3^) achievable. This technology, named acid-base flow battery (AB-FB), is based on the reversible water dissociation via bipolar membranes, and stores electricity in the form of the chemical energy of a pH gradient. It has received little attention so far; however, it has an interesting potential.

Pärnamäe et al. [8] present a perspective article with the state-of-the-art and latest developments of the AB-FB. The technology has already been demonstrated at the laboratory scale with maximum energy density and power density in discharge of ~10 kWh m^−3^ and ~17 W m^−2^ membrane, respectively. The discharge power is still limited by delamination issues that may damage irreversibly the bipolar membrane at high discharge currents. Multi-stage scenarios are simulated by a process model, highlighting that, to reach AB-FB applications on the kW–MW scale, further studies should focus on (i) the optimization of the plant design and (ii) the development of improved membranes. These advancements are crucial to reduce the membrane area and the solutions’ volume and to increase the round-trip efficiency. Experimental testing of the first 1 kW/7 kWh pilot plant is currently ongoing. A techno-economic analysis is performed for the pilot plant and for a first-of-a-kind commercial plant of 100 kW/700 kWh. With an increase in power density up to 30–40 W m^−2^ and with a projected cost of membranes of 100 € m^−2^ per triplet, the commercial unit would attain a unitary cost of 470 € kWh^−1^. The development of improved and cheaper components (especially bipolar membranes) and of optimized designs can lead the AB-FB technology to play a role in future systems of energy storage.

In conclusion, the papers in this Special Issue illustrate how experimental activities and modelling approaches can be used at several levels providing a significant contribution to the progress of electromembrane processes through the development of high-performance membranes and of improved engineered systems.
